# A straightforward spectral emissivity estimating method based on constructing random rough surfaces

**DOI:** 10.1038/s41377-023-01312-1

**Published:** 2023-11-07

**Authors:** Zezhan Zhang, Mengchao Chen, Lichuan Zhang, Hongzu Li, Hairui Huang, Zilong Zhang, Peifeng Yu, Yi Niu, Shan Gao, Chao Wang, Jing Jiang

**Affiliations:** 1 Clean Energy Materials and Engineering Center, School of Integrated Circuit Science and Engineering, University of Electronic Science and Technology of China, Chengdu, China; 2 College of Information and Communication Engineering, Harbin Engineering University, Harbin, China; 3 Department of Precision Instrument, Tsinghua University, Beijing, China

**Keywords:** Infrared spectroscopy, Imaging and sensing

## Abstract

Spectral emissivity is an essential and sensitive parameter to characterize the radiative capacity of the solid surface in scientific and engineering applications, which would be non-negligibly affected by surface morphology. However, there is a lack of assessment of the effect of roughness on emissivity and a straightforward method for estimating the emissivity of rough surfaces. This paper established an estimating method based on constructing random rough surfaces to predict rough surface (Geometric region) emissivity for metal solids. Based on this method, the emissivity of ideal gray and non-gray body surfaces was calculated and analyzed. The calculated and measured spectral emissivities of GH3044, K465, DD6, and TC4 alloys with different roughness were compared. The results show that the emissivity increases with the roughness degree, and the enhancement effect weakens with the increase of roughness or emissivity due to the existing limit (emissivity ε = 1.0). At the same time, the roughness would not change the overall spectral distribution characteristics but may attenuate the local features of the spectral emissivity. The estimated results are in good agreement with the experimental data for the above alloys’ rough surfaces. This study provides a new reliable approach to obtaining the spectral emissivity of rough surfaces. This approach is especially beneficial for measuring objects in extreme environments where emissivity is difficult to obtain. Meanwhile, this study promotes an understanding of surface morphology’s effect mechanism on emissivity.

## Abstract (web summary)

Flow diagram of the straightforward spectral emissivity estimating method for rough surfaces of metal solids based on constructing random rough surfaces

## Graphical abstract

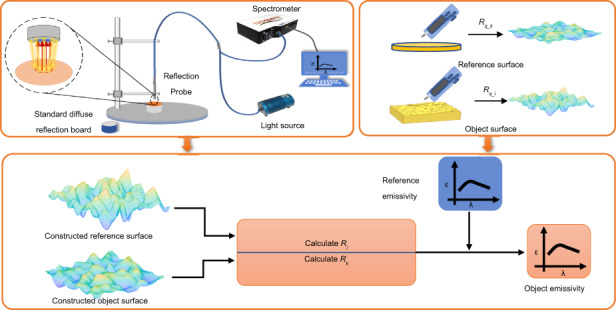

## Introduction

Spectral emissivity is an essential and sensitive parameter to characterize the radiative ability of solid surfaces, which plays an important role in scientific and engineering applications such as heat transfer analysis, infrared camouflage, non-contact radiation thermometry, semiconductor devices feature characterization, and remote sensing^1–5^. Accurate spectral emissivity knowledge is significant for highly temperature-dependent applications, such as thermometry for gas turbines and aero-engine operating status monitoring and thermal radiation modulating for infrared camouflage^6,7^. However, the spectral emissivity of a solid surface is not constant, which is influenced by temperature, surface composition, coatings, oxidation, and surface morphology^8–11^. Therefore, the spectral emissivity of a specific material solid has no unique and widely recognized value, and only reference values are provided in product brochures and literature.

Experimental measurement is always employed to obtain the emissivity of object surfaces in engineering applications^12^. Spectral emissivity characteristics of many alloys have been experimentally researched, including nickel-base alloy, aluminum alloy, and titanium alloy. In these works, the spectral emissivity of alloys at different temperatures and oxidation states was measured, and the influence mechanism of composition, temperature, oxidation, and surface morphology on spectral emissivity was qualitatively analyzed^13–18^. Moreover, some mathematical models of the emissivity-oxidation and emissivity-temperature are established to estimate the spectral emissivity variation of object surface at different states. These models help to select or predict the appropriate emissivity for radiation thermometry and heat transfer calculations according to conditions like surface composition, temperature, and oxidation degree^19–24^. However, these works paid insufficient attention to quantitatively investigating surface morphology’s effect. It still can be found that even with the same surface composition, temperature, and oxidation, the spectral emissivity of solid surfaces with different morphology varies. Because the rough degree of the alloy surface would affect the effective radiation area essentially, thereby affecting the surface radiative capacity.

To study the non-negligible effect of surface morphology on spectral emissivity, researchers have attempted to establish theoretical models describing the effect of surface morphology on emissivity. Moreover, they explored approaches for estimating emissivity from surface morphology. The function between emissivity and surface morphology was modeled using geometric optics (GO), and it was used to determine the emissivity of gray-diffuse rough surfaces with thermal and optical homogeneity^25–27^. Moreover, this model was experimentally proved effective in researching the effect on emissivity casing by alloy surface roughness^28^. In addition, some improved models were established to calculate the unknown regular rough surface emissivity with known roughness parameters^29–31^. However, since the key parameter roughness factor is difficult to obtain, these methods are inconvenient to implement for roughness randomly distributed surfaces. In detail, the roughness factor of a randomly rough surface would be manually obtained from the profilogram^32,33^, which may introduce artificial errors. So far, there is no easy-to-implement method for estimating the emissivity variety of surfaces with different roughness states for engineering applications.

This study provides a straightforward spectral emissivity estimating method (SEEM) for rough surfaces of metal solids based on constructing random rough surfaces (CRRS). This method could conveniently calculate the spectral emissivity of unknown surfaces with different roughness states on the same metal solids. Based on this method, we calculated and analyzed the roughness effect on the surface emissivity of gray and non-gray bodies, which are common hypothetical ideal physical models. This method is further verified by comparing the spectral emissivity of GH3044, K465, TC4, and DD6 alloys with different roughness at room temperatures. It should be noted that spectral emissivity has directional characteristics, and the directional spectral emissivity ε of a solid surface can be approximated by normal spectral emissivity ${\varepsilon }_{n}$ in engineering^34^. Therefore, the normal spectral emissivity was adopted as spectral emissivity to study the radiative characteristics of rough surfaces in this study.

## Results

### Methodology for spectral emissivity estimation based on CRRS

**Fig. 1:**
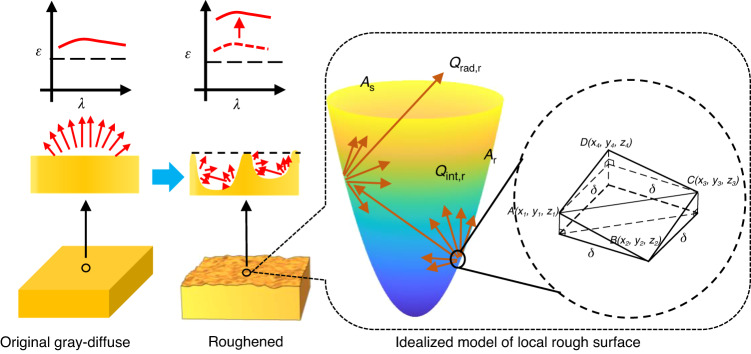
The radiative energy transfer model of the local area of rough surface

Rough surfaces can be divided into the spectral region, intermediate region, and geometric region based on optical roughness (${R}_{q}/\lambda$), which is represented by the ratio of root-mean-square (RMS) surface roughness ${R}_{q}$ to wavelength λ. In geometric region (${R}_{q}/\lambda > 1.0$), emissivity is highly sensitive to the detailed surface geometry, and diffraction effects are ignored here because the effects of roughness on emissivity are quite large compared to those of wavelength^31^. As shown in Fig. 1, a simple yet effective method for determining the emissivity of grey-diffuse rough surfaces with thermal and optical homogeneity was developed using GO^28^. According to the correlation of emissivity with surface roughness parameters (see Supplementary Information Section 1), for a surface in a thermal equilibrium state ($\varepsilon =\alpha$), emissivity can be expressed as

1 $${\varepsilon }_{{\rm{r}}}={[1+(\frac{1}{{\varepsilon }_{{\rm{s}}}}-1)R]}^{-1}$$

In Eq. (1), ${\varepsilon }_{{\rm{r}}}$ is the emissivity of the equivalent surface *A*_s_, and ${\varepsilon }_{{\rm{s}}}$ is the emissivity of *A*_r_, and *R* is the roughness factor.

Equation (1) can also be used to relate the emissivities of surfaces with different roughness factors ${R}_{i}$ and ${R}_{k}$ but the same materials by

2 $${\varepsilon }_{i}={[1+(\frac{1}{{\varepsilon }_{k}}-1)\frac{{R}_{i}}{{R}_{k}}]}^{-1}$$

Equation (2) shows the relationship between the roughness factor and the spectral emissivity between two rough surfaces with the same material. It provides the probability of calculating the spectral emissivity of an object’s rough surface from its roughness factor. In other words, if a surface with known emissivity and roughness factor *R* was selected as a reference, its roughness factor could calculate the spectral emissivity of other target surfaces with the same material. The main challenge to implementing this is conveniently obtaining the roughness factors ${R}_{i}$ and ${R}_{k}$ of the reference and object surfaces.

**Fig. 2:**
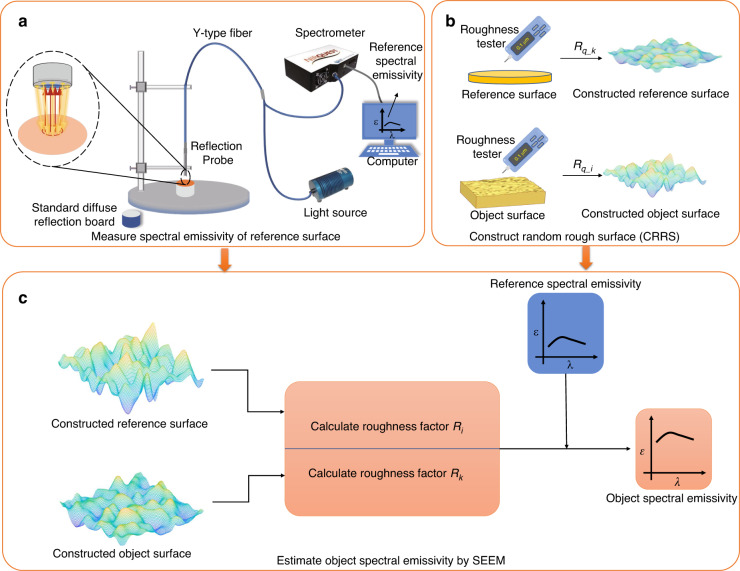
Flow diagram of the SEEM by CRRS for rough surfaces. **a** Measure the spectral emissivity of a reference surface by reflection method (step 1); **b** Construct the random rough surface by measured *R*_*q*_ (step 2); **c** Calculate the spectral emissivity of object surface (step 3)

It can be seen from Eq. (7) that the roughness factor *R* is the ratio of the effective radiation area to the actual area for a rough surface (see Supplementary Information Section 1). So, this study obtains the roughness factor by CRRS to address this challenge. Garcia and Stoll outlined the method to generate simulation 2D random rough surfaces^35^, and the implementation details in this paper are presented in the Supplementary Information Section 2. Combining Eqs. (2) and 2D rough surface generation, we propose a method to calculate the emissivity of the geometric regions (${R}_{q}/\lambda > 1.0$) of rough surfaces by CRRS. The main steps are shown in Fig. 2. The first step is to measure the spectral emissivity of the reference surface (Fig. 2a). The second step is to measure the *R*_*q*_ of the reference and object surfaces, and then construct the corresponding simulated 2D random rough surface (Fig. 2b). The parameter *R*_*q*_ can be easily obtained by using a roughness meter or an optical profiler. The last step is to calculate the roughness factors *R*_*i*_ and *R*_*k*_ from the constructed rough surfaces and then obtain the spectral emissivity of the object surface (Fig. 2c). It should be noted that common methods for measuring spectral emissivity include the calorimetric method, reflectance measurements method, multispectral method, and direct method. The reflection method is adopted and shown in Fig. 2a because it suits the implementation for room temperature objects.

**Fig. 3:**
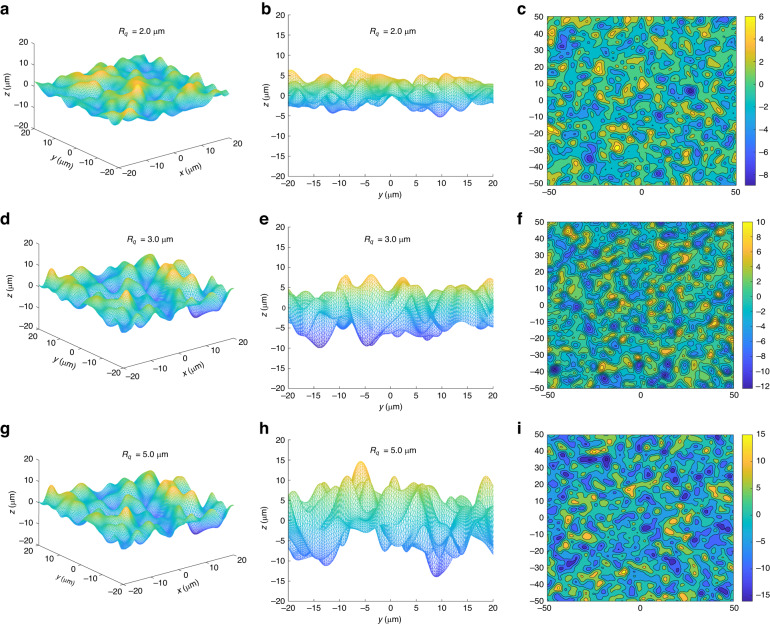
Generated 2D random rough surfaces with different *R*_*q*_. **a**–**c** *R*_*q*_ = 2.0 μm; **d**–**f** *R*_*q*_ = 3.0 μm; **g**–**i** *R*_*q*_ = 5.0 μm

Figure 3 shows the constructed 2D random rough surfaces with different *R*_*q*_ (2.0 μm, 3.0 μm, and 5.0 μm). All the surface profiles exhibit irregular roughness characteristics, and the degree of roughness is positively correlated with *R*_*q*_. The rough surface with *R*_*q*_ 2.0 μm has the slightest degree of random fluctuation, while the rough surface with *R*_*q*_ 5.0 μm has the most significant degree of fluctuation. This proves that *R*_*q*_ is one of the main factors affecting the roughness of the numerically simulated random rough surface, and we can control the rough degree of the generated random rough surface through *R*_*q*_. Thus, the area ${A}_{{\rm{r}}}$ of these surfaces were obtained according to Eq. (7) of the Supplementary Information Section 1. The areas ${A}_{{\rm{r}}}$ of the three random surfaces were 4.65 × 10^3^ μm^2^, 6.21 × 10^3^ μm^2^, and 9.74 × 10^3^ μm^2^ respectively. It can be qualitatively inferred that the rougher surface has a stronger radiation emission capacity than the smooth surface due to the larger effective radiation surface area.

It should be noted that the constructed rough surfaces with the same *R*_*q*_ were not the same, and the area and roughness factors *R* distribution of 10 random rough surfaces generated based on the same *R*_*q*_ (2.0 μm, 3.0 μm, and 5.0 μm) is shown in Fig. S1 (see Supplementary Information Section 3.1). In this study, the median of the roughness coefficient *R* of 10 constructed surfaces was selected for calculation.

### Spectral emissivity calculation of gray body and non-gray models

Ideal gray body and non-gray models are commonly used in radiation analysis and calculations. The gray body model means that the emissivity of an object is a constant value without spectral characteristics, and the practical object is often treated as a gray body within a specific spectral range to simplify the calculation in engineering. In contrast, the non-gray model refers to real objects with non-neglected spectral emissivity characteristics. The relationship between surface roughness and the two kinds of models remains elusive. So, hypothetical gray body emissivity 0.2–0.9 and four spectral-emissivity models used in multispectral radiation thermometry (MRT) were selected as objects to calculate and analyze the effect of roughness on the gray body and non-gray type surfaces.

**Fig. 4:**
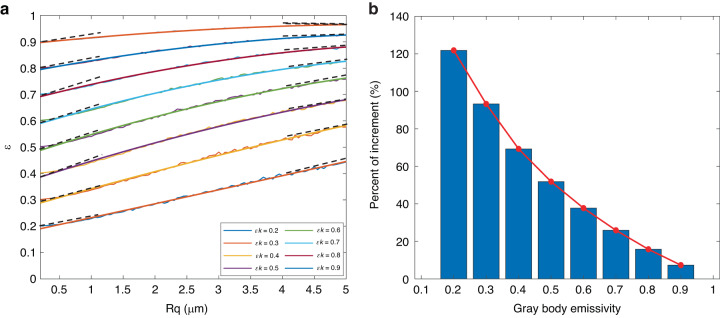
The variety of gray body emissivity caused by roughness. **a** The change of gray body emissivity in *R*_*q*_ ranges from 0.2 to 5.0 μm; **b** The relative increment at *R*_*q*_ = 5.0 μm of gray body emissivity for different initial emissivity

Figure 4 shows the result of the emissivity calculation of the generated gray body surface with different *R*_*q*_ (0.2 μm–5.0 μm). In the calculation, the reference surfaces emissivity ${\varepsilon }_{k}$ ranged from 0.2 to 0.9 with step 0.1, and the original *R*_*q*_ of the reference surface was set as 0.1, ignoring the spectral properties of emissivity.

It can be observed from Fig. 4a that the emissivity of the gray body is positively correlated with *R*_*q*_. In the range of *R*_*q*_ at 0.2–5.0 μm, the emissivity of all surfaces increases with *R*_*q*_. The initial emissivity of 0.2, 0.5, and 0.9 were increased to 0.44, 0.76, and 0.96 when *R*_*q*_ was 5.0 μm. At the same time, the gradient of emissivity increases gradually decreases with the increase of *R*_*q*_. At the initial emissivity above 0.4, the relationship between emissivity and *R*_*q*_ presents a nonlinear characteristic, and the increasing gradient of emissivity when *R*_*q*_ = 0.2 μm was smaller than *R*_*q*_ = 5.0 μm. The relative increase in emissivity at *R*_*q*_ = 5.0 μm was calculated and shown in Fig. 4b. The relative increments of initial emissivity due to roughness decreased as the initial emissivity increased. When the initial emissivity was 0.2, 0.5, and 0.9, the relative increment of emissivity at *R*_*q*_ = 5.0 μm was 120.30%, 52.01%, and 7.35%, respectively.

In the real scenes where the object cannot be regarded as a gray body, the spectral characteristics of the object’s emissivity need to be pre-obtained as an important reference. So, four spectral emissivity models commonly used in multispectral radiometric thermometry (MRT) were chosen as the research object, and their variety with different rough degrees was calculated. As shown in Table 1, linear, quadratic, sinusoidal, and exponential types of spectral-emissivity models were chosen, and a surface with *R*_*q*_ = 0.10 μm was set as the reference, then *R*_*q*_ was set to 2.0 μm, 3.0 μm, and 5.0 μm respectively to simulate an increase in surface roughness degree. The range of spectrum was set to 1.3 μm–2.3 μm, which was consistent with later superalloy spectral emissivity measurement experiments.

**Table 1:** Emissivity models and parameters

Type	Mathematical function	*R*_*q*_ (μm)	Range of spectrum
Linear	$\varepsilon =a\lambda +b$	2.0, 3.0, 5.0	1.3 μm–2.3 μm
Quadratic	$\varepsilon =a{\lambda }^{2}+b\lambda +c$	2.0, 3.0, 5.0	1.3 μm–2.3 μm
Sinusoidal	$\varepsilon =a[\sin \left(b\lambda +c\right)]+d$	2.0, 3.0, 5.0	1.3 μm–2.3 μm
Exponential	$\varepsilon =a[\exp \left(b\lambda +c\right)]+d$	2.0, 3.0, 5.0	1.3 μm–2.3 μm

**Fig. 5:**
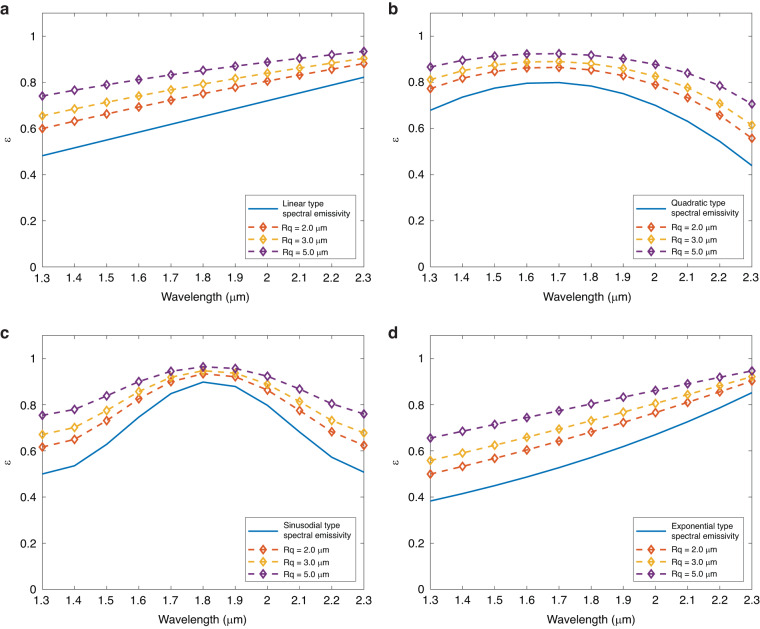
Spectral emissivity variety induced by rough surfaces. **a** Linear type spectral emissivity; **b** Quadratic type spectral emissivity; **c** Sinusoidal type spectral emissivity; **d** Exponential type spectral emissivity

Figure 5a–d shows the calculation results of the four spectral-emissivity models. The solid blue line in Fig. 5a–d is the initial emissivity of each spectrum-emissivity model, and the dotted lines represent the spectral emissivity of surfaces with *R*_*q*_ of 2.0 μm, 3.0 μm, and 5.0 μm, respectively. It could be seen that with the *R*_*q*_ increases, the spectral-emissivity of each model was greater than the initial emissivity, and the increase of *R*_*q*_ = 5.0 μm had the largest increment. In addition, the change of roughness *R*_*q*_ did not affect the type of spectral-emissivity model. The types of spectral-emissivity in Fig. 5a–d were still linear, quadratic, sinusoidal, and exponential, even though the curve parameters were changed.

The roughness increases the surface spectral emissivity, which is not a simple linear increase. It can be observed from Fig. 5c that at the wavelength of 1.8 μm, the increase in roughness to emissivity is smaller than that at other wavelengths like 1.3 μm. Likewise, the roughness did not change the surface wavelength-spectral emissivity model type. In Fig. 5a–d, even if the model parameters changed, the spectral-emissivity model type kept the same as the original. The same went for the positive or negative correlation between wavelength and emissivity. It is worth noting that roughness increases the emissivity of a surface but does not make the emissivity of a solid surface exceed the emissivity of a black body (ε = 1.0).

Figure S2 shows the relative increment of spectral-emissivity models caused by *R*_*q*_ (see Supplementary Information Section 3.2). For the four models, within 1.3 μm–2.3 μm, a large *R*_*q*_ would cause a larger increase in spectral emissivity, and the increase caused by *R*_*q*_ = 5.0 μm was the largest, followed by *R*_*q*_ = 3.0 μm and *R*_*q*_ = 2.0 μm being the smallest. The relationship type between the increment and the wavelength is the same as the original emissivity, and the curves of the increment versus wavelength in Fig. S2a–d were linear, quadratic, sinusoidal, and exponential, respectively. However, emissivity increment and wavelength monotonicity were opposite to the original emissivity. For example, in Fig. S2a, the increment decreases linearly with wavelength, while the original emissivity increases linearly, and the phenomenon was also observed in Fig. S2b–d.

### Normal spectral emissivity calculation of superalloys

The performance of this approach on real random rough surfaces was evaluated based on the real alloys. The *R*_*q*_ of GH3044, K465, DD6, and TC4 alloys with different rough states at 300 K was measured, and spectral emissivity calculation was carried out based on the measurement results. The reason for choosing these alloys was that they are commonly used to manufacture thermal components of aero-engines and gas turbines. Their spectral emissivity has important implications for heat treatment in manufacturing. An optical profiler obtained the *R*_*q*_ of the polished superalloys, and the result are shown in Table S1 (see Supplementary Information Section 3.3). The *R*_*q*_ of the GH3044 alloy surface ranges from 0.76 to 3.12, the *R*_*q*_ of the K465 alloy surface is 0.59 to 3.70, the *R*_*q*_ of the DD6 alloy surface ranges from 0.45 to 3.60, and The *R*_*q*_ of the TC4 alloy surface ranges from 0.48 to 3.38.

Samples with smoother surfaces have smaller *R*_*q*_, and the sample G0, K0, T0, and D0 with *R*_*q*_ < 1.0 in Table S1 were selected as references. At the same time, the spectral emissivity of these reference surfaces was obtained using the reflection method as the reference spectral emissivity. According to the flow chart in Fig. S3 (see Supplementary Information Section 3.4), the spectral emissivities of the alloy surfaces with different *R*_*q*_ were estimated.

A linear modification was adopted to calculate the spectral emissivity of the rough surfaces to improve the reliability of the approach^31^. The modified normal spectral emissivity calculating function based on Eq. (2) is

3 $${\varepsilon }_{r}=a{\left[1+(\frac{1}{{\varepsilon }_{k}}-1)\frac{{R}_{i}}{{R}_{k}}\right]}^{-1}+b$$

In Eq. (3), a and b are correction factors, which can be obtained by linear regression (*a* = 0.425 and *b* = 0.590).

**Fig. 6:**
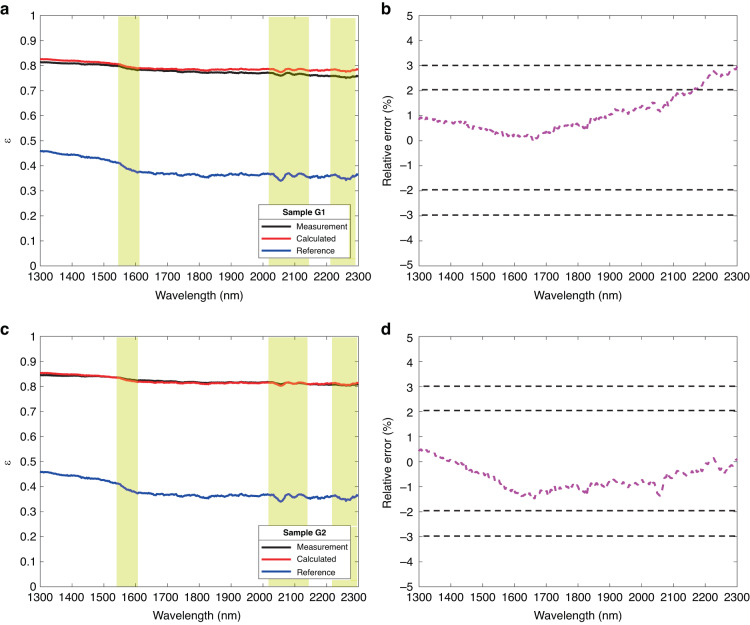
Spectral emissivity obtained by SEEM and measuring the GH3044 samples. **a** Spectral emissivity calculation and measurement results for a surface with *R*_*q*_ = 2.10 μm; **b** Relative error in calculation and measurement of surface spectral emissivity for *R*_*q*_ = 2.10 μm; **c** Spectral emissivity calculation and measurement results for a surface with *R*_*q*_ = 3.12 μm; **d** Relative error in calculation and measurement of surface spectral emissivity for *R*_*q*_ = 3.12 μm

The emissivity calculation results and relative errors of the GH3044 alloy are shown in Fig. 6. Figure 6a, b and c, d are the calculation results of G1 (*R*_*q*_ = 2.10 μm) and G2 (*R*_*q*_ = 3.12 μm), respectively. The solid blue line represents the spectral emissivity of the G0 (*R*_*q*_ = 0.76 μm) was set as a reference, which is less than 0.5 in the wavelength of 1.3 μm–2.3 μm. The spectral emissivity of samples G1 and G2 was greater than that of the surface of G0 (*R*_*q*_ = 0.76 μm). At the same time, the spectral emissivity of the surface with different *R*_*q*_ has the same trend characteristics. For example, the turning points at 1.60 μm and 1.80 μm appeared on the spectral emissivity of all the sample surfaces. From Fig. 6b, d, the calculated spectral emissivity is almost consistent with the measured results, with a maximum relative error of less than ± 3%.

**Fig. 7:**
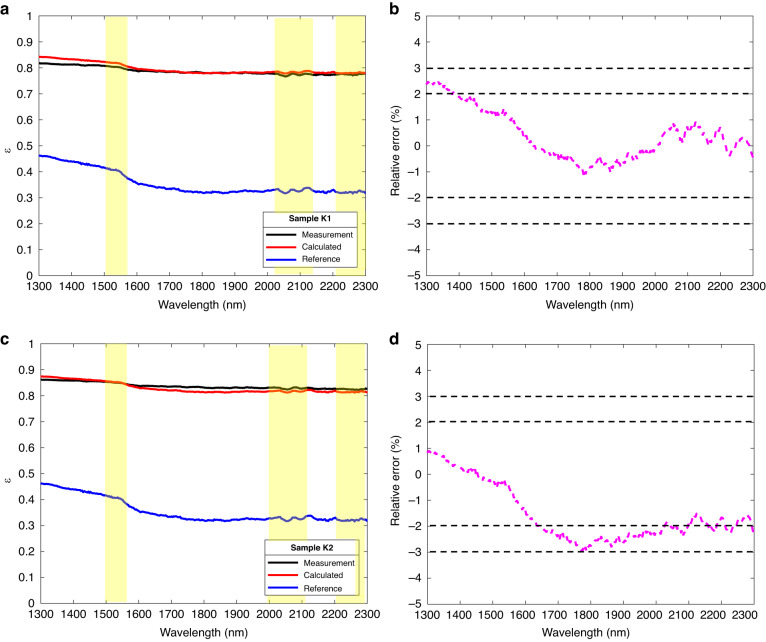
Spectral emissivity obtained by SEEM and measuring the K465 samples. **a** Spectral emissivity calculation and measurement results for a surface with *R*_*q*_ = 2.39 μm; **b** Relative error in calculation and measurement of surface spectral emissivity for *R*_*q*_ = 2.39 μm; **c** Spectral emissivity calculation and measurement results for a surface with *R*_*q*_ = 3.70 μm; **d** Relative error in calculation and measurement of surface spectral emissivity for *R*_*q*_ = 3.70 μm

Figure 7 shows the emissivity calculation results and relative errors of the K465 alloy. Figure 7a–d are the calculation results of samples K1 (*R*_*q*_ = 2.39 μm) and K2 (*R*_*q*_ = 3.70 μm), respectively. The solid blue line is the spectral emissivity of the K0 (*R*_*q*_ = 0.59) reference surface, close to the spectral emissivity of G0 (less than 0.5 in the wavelength of 1.2 μm–2.3 μm). The spectral emissivities of K1 (*R*_*q*_ = 2.10 μm) and K2 (*R*_*q*_ = 3.12 μm) were greater than that of the K0 (*R*_*q*_ = 0.76 μm) because of the larger rough degree. Similar to GH3044 alloy, the spectral emissivity of the surface with different *R*_*q*_ has the same trend characteristics, and the turning point was at 1.6 μm. From Fig. 7b, d, the calculated spectral emissivity is almost consistent with the measured results, and the maximum relative error was less than ± 3% too.

Figures S4–S5 show the emissivity calculation results and relative errors of the TC4 and DD6 alloy (see Supplementary Information Section 3.5 and 3.6). The spectral emissivity obtained by this method is also consistent with the experimental value for those two alloys. The maximum relative error of TC4 alloy is less than ± 4%, while the maximum relative error of TC4 alloy is less than ± 5%.

Figures 6 and 7 and S4S5 show the comparison between the theoretically calculated result and experimental result of GH3044, K465, DD6, and TC4 alloys with rough surfaces at 300 K. It can be observed that the calculated and experimentally measured spectral emissivity of the four alloys were consistent with the overall trend at 1.3 μm–2.3 μm. In addition, the spectral emissivity of the surface with greater roughness is higher than that of the reference surface. This phenomenon was consistent with the spectral emissivity calculation of the spectral-emissivity model. Combining the results of these four alloys, it can be found that the spectral emissivity estimation method based on CRRS can estimate the spectral emissivity of GH3044, K465, DD6, and TC4 alloys, and the calculated emissivity was consistent with the real emissivity characteristic with relative error within ± 5% at the wavelength 1.3 μm–2.3 μm.

To verify the effectiveness of SEEM in a larger wavelength range, we selected the metal cobalt’s roughness and spectral emissivity data from Liu’s work for verification^31^. The data of sample 3, sample 11, and sample 13 in this reference literature were selected. Using sample 3 as a reference sample, we calculated the spectral emissivity of samples 11 and 13 at 773 K, respectively. The results are shown in Fig. S6 (see Supplementary Information Section 3.7), and it can be seen that the overall spectral distribution trend of the measured and calculated spectral emissivity of metal cobalt in the range of 3.0 μm–20.0 μm was consistent. Still, the relative error of the calculation was more than ± 10%. We inferred it was due to the error induced by obtaining data from the literature using the GetData software.

## Discussion

We explored the effect mechanism of roughness on surface emissivity based on the SEEM. The results show that the roughness degree could enhance the radiative ability of solid surfaces overall. This enhancement effect weakens with the increase of roughness or initial emissivity. According to Kirchhoff’s law, the absorptivity of an opaque object is equal to the emissivity (α(λ) = β(λ)) in thermodynamics^36^. Therefore, the surface absorption of thermal radiation could explain the emissivity rising phenomenon caused by roughness shown in Figs. 4 and 5 for the gray and non-gray bodies. Roughness increases the effective area of the surface by enhancing the degree of wrinkling, thereby enhancing the ability of the surface to capture and absorb incident radiation energy, thus overall increasing the surface’s absorption rate α of radiation.

However, the increased emissivity caused by roughness would not equal or exceed 1.0. Because the upper limit of this radiation absorption enhancement is that the surface absorbs almost all the incident radiation, which means the surface radiation absorption is close to that of an ideal blackbody (α = ε = 1.0). Therefore, it can be deduced that in Figs. 4 and 5, the increased emissivity limit of the gray and non-gray bodies is close to 1.0. The effect of roughness on surface emissivity enhancement weakens with the increase of roughness and initial emissivity of materials, which is also due to the limit of surface absorption capacity. Because of the existence of this limit, when the emissivity ε (absorption rate α) is closer to 1.0, the influence factors such as roughness and original emissivity (absorption rate α) that can enhance the surface radiation (absorption) ability are weaker to meet the energy conservation law. So, the spectral distribution of the emissivity increment is opposite to the original spectral characteristic.

In addition, the roughness would not change the overall spectral distribution characteristics but may attenuate the local features of the spectral emissivity. As the measurement results of GH3044, K465, DD6, and TC4 alloys shown in Figs. 6–7 and Figs. S4–S5, the overall trend of the increased spectral emissivity caused by roughness decreases with the wavelength, which is consistent with the spectral characteristics of the initial reference emissivity. This phenomenon occurs due to the weakening of the diffraction effect in the geometric region (${R}_{q}/\lambda > 1.0$). In the geometric region, the emissivity is highly sensitive to the surface morphology ignoring the diffraction effect, and the effect of roughness on emissivity is much greater than that on wavelength. So, the roughness has a rare effect on the spectral properties of the emissivity. Nevertheless, the roughness may attenuate the local features of the spectral emissivity, as shown in the yellow region where the difference between peaks and troughs was reduced.

Very few studies have systematically investigated the effect of surface roughness on spectral emissivity. Most available studies have been conducted to measure and analyze the effect of temperature, oxidation, and morphology on normal spectral emissivity. However, their findings seem consistent with our study. For example, Liu et al. showed that the amplitude of the normal spectral emissivity of DD6 alloy (3.0 μm–20.0 μm), cobalt (3.0 μm–20.0 μm), and E235B low carbon structural steel (2.0 μm–14.0 μm) increased with the roughness at the observed wavelength^31,37,38^. However, no representation or explanation of the spectral distribution trend was provided. Cheng et al. observed the normal emissivity of graphite increasing with surface roughness at 5.5 μm–17.0 μm^39^. He attributed the emissivity increase to small facets of the surface enhancing the surface’s ability to scatter and reflect electromagnetic waves, and pores be seen as the blackbody increasing the radiative capability of the surface. In Cheng’s study, even though it was not mentioned, it could still be observed that the normal spectral emissivity overall increased with the roughness without the spectral distribution varying, and the feature detail of spectral emissivity at 8.1 μm and 10.0 μm was weakened.

To compare the performance of methods using surface roughness to calculate emissivity, we summarize the existing methods in Table 2. It should be noted that the maximum relative errors of the previous methods were estimated according to the data obtained from the references. Hence, they are not rigorously equal to the real values.

**Table 2:** The methods for calculating emissivity from surface roughness parameters

Emissivity calculation method	Main formula	Means of obtain the key roughness factor *R*	Application scope	Performance in typical researches (Maximum relative error of experimental and theoretical emissivity values)
Original Agababov’s method	${\varepsilon }_{r}={[1+(\frac{1}{{\varepsilon }_{s}}-1)R]}^{-1}$, $R={(1+{\pi }^{2}{n}^{2}{\sigma }^{2})}^{-1}$	Manually calculate *R* from the 1D surface profilogram.	Surface with randomly distributed roughness	Aluminum alloy AL7075 in C.-D Wen’s research^28^: beyond ± 30% without the effect of CO_2_ and H_2_O absorption for AL 7075 samples roughened with 6 μm and 14 μm grit paper.
Improved model based on Agababov’s roughness function	${\varepsilon }_{r}={[1+(\frac{1}{{\varepsilon }_{s}}-1)R]}^{-1}$, $R={\left[1+4{(\frac{H}{L})}^{2}\right]}^{-1}={\left[1+4{(\frac{{R}_{a}}{{R}_{s}})}^{2}\right]}^{-1}$	Use the measured *R*_*a*_ and *R*_*s*_ to replace *H* and *L* respectively, then calculate *R*.	Surface with regularly distributed roughness	Cobalt in Kun Yu’s research^31^: less than ± 10% at the wavelength of 3.0 μm–15.0 μm for cobalt samples roughened by 180, 400, and 1200 grit papers.
Method based on CRRS	${\varepsilon }_{i}={[1+(\frac{1}{{\varepsilon }_{k}}-1)\frac{{R}_{i}}{{R}_{k}}]}^{-1}$	Obtain *R* from constructing 2D random rough surfaces.	Surface with randomly distributed roughness	K465, GH3044, DD6, and TC4 alloys of this paper: less than ± 5% for alloy surface roughened by 36, 80, 240, and 1200 grit papers.

As shown in Table 2, C.-D Wen used the original Agababov’s method to calculate the spectral emissivity of Al-7075 alloys with different rough surfaces. The maximum relative error was beyond ± 30% without the effect of CO_2_ and H_2_O absorption. In the original Agababov’s method, the core parameter *R* is manually calculated through the surface 1D profilogram. Kun Yu proposed an improved method based on the original Agababov’s method and calculated the spectral emissivity of rough cobalt surfaces with regularly distributed roughness. The maximum relative error was less than ± 10% which was better than the original Agababov’s method. In this improved method, the key roughness factor *R* could be conveniently calculated without the exact surface profilogram because a hypothesis (the mean arithmetic deviation *R*_*a*_ and average single-peak spacing *R*_*s*_ were equal to *H* and *L*). However, as the authors mentioned, this method was limited to the surface with regular roughness distribution because this hypothesis would lead to a certain degree of error. In this paper, the spectral emissivity of GH3044, K465, DD6, and TC4 alloys with different rough surfaces were calculated using the SEEM based on CRRS, and the maximum relative error was less than ± 5%. In this method, the roughness factor *R* was directly calculated from the computationally constructed 2D random rough surfaces without surface profilogram and hypothesis, which is convenient for implementation for engineering applications.

In conclusion, we have proposed and experimentally demonstrated a straightforward SEEM by CRRS for unknown rough surfaces (${R}_{q}/\lambda > 1.0$) of metal solids. Using this approach, we calculated and evaluated the effect of roughness on the emissivity of gray and non-gray body-type surfaces. We found that the emissivity increases with the roughness. Still, the limit of emissivity increase was 1.0 since the emissivity enhancement effect decreases with roughness and original emissivity. In addition, we compared the measured and calculated emissivity of GH3044, K465, DD6, and TC4 alloys with different roughness to evaluate the effectiveness of this approach. We found that the maximum relative error of calculation was ± 5%. At the same time, the effect of roughness on the normal spectral emissivity of these four alloys is consistent with the conclusion of non-gray bodies obtained by calculation. It is worth noting that this study paves a new way to obtain the spectral emissivity of rough surfaces and promotes the understanding of surface morphology’s effect mechanism on emissivity, which would meet the challenges in scientific and many engineering applications.

## Materials and methods

### Sample preparation and surface R_q_ measurement

In this paper, GH3044, K465, DD6, and TC4 alloys were selected as the research objects, and the compositions of the four alloys at room temperature are listed in Table S2 (see Supplementary Information Section 3.8). In this paper, three procedures were used to prepare samples. First, three standard cylindrical samples with a diameter of 17 mm and a height of 10 mm were prepared using these alloys by a machinery factory. Then, one surface of each sample was manually polished according to the abrasive paper specifications shown in Table S1 to obtain surfaces with different random roughness. Finally, the polished samples were cleaned with acetone, ultrasonically cleaned in ethanol for 5 minutes, and dried in a drying oven. After sample preparation, the *R*_*q*_ of all the polished surfaces was measured using TR100 roughness tester, and the results are listed in Table S1 (see Supplementary Information Section 3.3).

### Measurement of spectral emissivity by reflectance method

The normal spectral emissivity of the samples at 300 K was measured by the reflectance method. The principle is previously measuring the sample’s reflectance $\rho \left(\lambda \right)$, and then calculating the emissivity $\varepsilon \left(\lambda \right)$ by $\varepsilon \left(\lambda \right)=1-\rho \left(\lambda \right)$. In the measurement, the reflectance spectrum of the standard reflector was used as a reference, and the sample absolute reflectance is obtained by comparing the reflectance spectrum of the sample and reference from

4 $$\rho \left(\lambda \right)=\frac{S\left(\lambda \right)-D\left(\lambda \right)}{R\left(\lambda \right)-D\left(\lambda \right)}{\rho }_{r}(\lambda )$$

In Eq. (4), $S\left(\lambda \right)$ is the spectral intensity of the sample, $R\left(\lambda \right)$ is the spectral intensity of the standard reflector, $D\left(\lambda \right)$ is the background spectrum, and ${\rho }_{r}(\lambda )$ is the reflectance of the standard reflector.

The measurement device is shown in Fig. 2a, consisting of a near-infrared fiber optic spectrometer (Ocean Optics NIRQuest512-2.5), a tungsten-halogen lamp source (HL-2000-FHSA with a spectral range of 0.36–2.40 μm), a reflection probe (QP400-7-UV-VIS), a probe holder, a standard reflector and a computer. The reflection probe was a Y-shaped optical fiber whose detection end was placed vertically on the sample surface about 1 cm away. The signal end was divided into two optical fibers connected to the tungsten-halogen light source and the spectrometer. The light emitted by the halogen tungsten light source irradiates the sample surface through the reflection probe. The reflection probe receives and transmits the reflected light to the spectrometer simultaneously. The standard reflector with known spectral reflectivity was used as a standard reference for calibration, provided by the Institute of Optics and Electronics, Chinese Academy of Sciences. In addition, because of the low signal-to-noise ratio of the spectrometer at a wavelength less than 1.3 μm and above 2.3 μm, the limit of the spectral range was set to 1.3 μm–2.3 μm.

### Supplementary information

Supplementary Information for A straightforward spectral emissivity estimating method based on

## Data Availability

The data that support the findings of this study are available from the corresponding author on reasonable request.
