# Supplementary material for: A straightforward spectral emissivity estimating method based on constructing random rough surfaces
Source: Light Sci Appl. 2023 Nov 7;12:266. doi: 10.1038/s41377-023-01312-1 (PMC10630351; doi:10.1038/s41377-023-01312-1)
Supplement: Supplementary file 1 — Supplementary Information for A straightforward spectral emissivity estimating method based on [file 41377_2023_1312_MOESM1_ESM.docx]

Supplementary Information for

A straightforward spectral emissivity estimating method based on constructing random rough surfaces

Zezhan Zhang^1^, Mengchao Chen^1^, Lichuan Zhang^1^, Hongzu Li^1^, Hairui Huang^1^, Zilong Zhang^1^, Peifeng Yu^1^, Yi Niu^1,*^, Shan Gao^2,*^, Chao Wang^3^, Jing Jiang^1,*^

^1^ Clean Energy Materials and Engineering Center, School of Integrated Circuit Science and Engineering, University of Electronic Science and Technology of China, Chengdu 611371, China

^2^ College of Information and Communication Engineering, Harbin Engineering University, Harbin 150001, China

^3^ Department of Precision Instrument, Tsinghua University, Beijing 100084, China

^*^ Correspondence: [niuyi@uestc.edu.cn](mailto:niuyi@uestc.edu.cn ( +86) ; [gaoshan08@hrbeu.edu.cn](mailto:gaoshan08@hrbeu.edu.cn); [jiangj@uestc.edu.cn](mailto:jiangj@uestc.edu.cn) (+86-28-61831325)

1. **Additional details of the correlation of emissivity with surface roughness parameters**

As shown in Fig. 1, the local surface roughness was modeled as a depression in the area *A*_r_*,* since the radiation of the surface *A*_r_ will be partially intercepted by itself, a view factor $F_{r,r}$ has been defined as the fraction of the intercepted radiation after leaving surface *A*_r_. Then for the total radiated energy $Q_{int, r}$ emitted by *A*_r_, part of the energy $Q_{int, r}(1-F_{r,r})$ would leave the equivalent surface *A*_s_. In contrast, the remaining part $Q_{int, r}F_{r,r}$ would return to the solid surface *A*_r_ again.

Defining the surface absorption rate as $\alpha_{s}$, then a portion of the returned energy $Q_{int,r}F_{r,r}\alpha_{s}$ will be absorbed by the surface, while the other energy $Q_{\mathrm{int}}F_{r,r}({1-\alpha}_{s})$ continue to be reflected by *A*_r_. Similarly, part of the reflected energy $Q_{int,r}F_{r,r}({1-\alpha}_{s})(1-F_{r,r})$ will leave through *A*_r_ and the other part $Q_{int,r}F_{r,r}^{2}({1-\alpha}_{s})$ falls back to *A*_r_. After continually repeating the reflection and absorption of radiation energy, the total radiated energy leaving the equivalent surface *A*_s_ of the rough surface is

$Q_{rad,r}=Q_{int,r}(1-F_{r,r})[1+F_{r,r}(1-\alpha_{s})+F_{r,r}^{2}\left( 1-\alpha_{s} \right)^{2}+\ldots]$ (1)

In Eq. (1), $Q_{rad,r}$ is the radiant energy leaving the equivalent surface *A*_s,_ and $Q_{int,r}$ is the total radiant energy emitted by the actual surface *A*_r_*.* The expression in square brackets is a decreasing infinite geometric series, so Eq. (1) yields

$Q_{rad,r}=Q_{int,r}(1-F_{r,r})\frac{1}{1-(1-\alpha_{s})F_{r,r}}$ (2)

Assuming $\varepsilon_{s}$ is the emissivity of *A*_r_, then

$Q_{int,r}=\varepsilon_{s}E_{b}A_{r}$ (3)

Where *E*_b_ is the blackbody radiation.

Similarly, $\varepsilon_{r}$ is the emissivity of the equivalent surface *A*_s_, then

$Q_{rad,r}=\varepsilon_{r}E_{b}A_{s}$ (4)

Combining Eqs. (2) ~ (4), $\varepsilon_{r}$ can be obtained from

$\varepsilon_{r}=\varepsilon_{s}\frac{A_{r}}{A_{s}}(1-F_{r,r})\frac{1}{1-(1-\alpha_{s})F_{r,r}}$ (5)

According to Fig. 1**b**, the view factor $F_{r,r}$ is given by

$F_{r,r}=1-\frac{A_{s}}{A_{r}}$ (6)

and the roughness coefficient *R* by

$R=\frac{A_{s}}{A_{r}}=\frac{l^{2}}{\sum_{i=1}^{M} A_{ri}}$ (*i* = 1, 2… M) (7)

1. **Additional details of methodology for spectral emissivity estimation based on CRRS**

***2.1 Construction of random rough surfaces***

The root-mean-square (RMS) deviation *R_q_* is commonly used in engineering to describe the roughness of real surfaces comprehensively. So, we adopted *R_q_* to generate custom rough surfaces, from which we could calculate the roughness coefficient to obtain spectral emissivity. In the statistical method, rough surfaces can be described by height density function (HDF), autocorrelation function (ACF), and power spectral density (PSD)^1^. Equally, rough surfaces can be constructed manually based on these three functions.

A rough surface can be described as Eq. (8)

$z=f(x,y)$ (8)

where *z* is the contour height of point *(x, y)* on the surface relative to the plane.

The height density function (HDF) describes the height distribution characteristics of rough surfaces. The height distribution of many engineering surfaces satisfies a Gaussian distribution ^2^. Thus, this paper assumes that the height of the surface is distributed according to the Gaussian density function shown in Eq. (9)

$p\left( z \right)=\frac{1}{h\sqrt{2\pi}}exp[-\frac{z^{2}}{2h}]$ (9)

Where *h* is the theoretical root-mean-square (RMS) height equivalent to the actual measured parameter *R_q_*.

The autocorrelation function (ACF) describes the transverse characteristics of the rough surface, which is also the basic statistical parameter for characterizing the spacing characteristics of the rough surface profile. For a rough surface, the Gaussian autocorrelation function can be expressed as

$C\left( x,y \right)=h^{2}exp(-\frac{x^{2}}{{l_{x}}^{2}}-\frac{y^{2}}{{l_{y}}^{2}})$ (10)

where *l_x_* and *l_y_* are the correlation lengths in the *x* and *y* directions, respectively, and they are statistically equivalent to the mean period of the rough surface.

The rough surface topography can be considered the sum of the sine (cosine) waves at various frequencies in the spatial coordinate system, so the overall frequency composition of the roughness topography in Fourier space can be described by employing the power spectral density (PSD). The power spectral density (PSD) is usually defined as the Fourier transform of the autocorrelation function (ACF) and can be expressed as

$P\left( k_{x},k_{y} \right)=\frac{h^{2}l_{x}l_{y}}{4\pi}exp \left( -\frac{{k_{x}}^{2}{l_{x}}^{2}+{k_{y}}^{2}{l_{y}}^{2}}{4} \right)k=2\pi/\lambda$ (11)

The Fourier transform $F(k_{x},k_{y})$ of the random rough surface topography can be described by the power spectral density (PSD) by the following equation

$F\left( k_{x_{m}},k_{y_{n}} \right)=2\pi l\sqrt{P\left( k_{x_{m}},k_{y_{n}} \right)}\left\{ \begin{aligned} \frac{N\left( 0,1 \right)+iN\left( 0,1 \right)}{\sqrt{2}} \\ N\left( 0,1 \right) \end{aligned} \right. {m,n\neq0,\frac{N}{2} \atop m or n=0,\frac{N}{2}}$ (12)

where $k_{x_{m}}=\frac{2\pi m}{l}$ and $k_{x_{n}}=\frac{2\pi n}{l}$, and *l* is the length of the rough surface, *N* is the number of nodes meshed by random rough surfaces, and $N(0,1)$ is a Gauss random number with a mean of 0 and RMS of 1.0.

Then, the random rough surface topography can be obtained by taking a two-dimensional discrete Fourier inverse transformation of $F(k_{x},k_{y})$ by

$z=f\left( x,y \right)=\frac{1}{l^{2}}\sum_{m=-N/2}^{\frac{N}{2}-1} \sum_{n=-N/2}^{\frac{N}{2}-1} F(k_{x_{m}},k_{y_{n}})exp(ik_{x_{m}}x+ik_{y_{n}}y)$ (13)

It should be known that random rough surface generation parameters *l*, *N*, $l_{x}$ and $l_{y}$ are usually static parameters, while *h* is the variable parameter that determines the rough profile of the surface. The area edge length *l* was 50 $\mu m$, the number of nodes *N* was 200, and the correlation length $l_{x}$ and $l_{y}$ in the *y*-direction is both 2 $\mu m$. The root-mean-square (RMS) deviation *R_q_* (2.0 $\mu m$, 3.0 $\mu m$, and 5.0 $\mu m$) was the adjustable parameter to generate different random rough surfaces.

***2.2 Surface area calculation***

The area of the rough surface can be calculated by the microelement sum shown in Fig. 1. The microelement surface is composed of four points *ABCD,* the side length of the corresponding smooth surface is δ, and the coordinates of the four vertices are denoted as $A\left( x_{1},x_{2},x_{3} \right)$,$B\left( x_{1},x_{2},x_{3} \right)$,$C\left( x_{1},x_{2},x_{3} \right)$ and$D\left( x_{1},x_{2},x_{3} \right)$, respectively. When using the conventional method to calculate the area, the side lengths of $\Delta ABC$ and $\Delta ACD$ can be calculated according to the vertex coordinates and the side length δ, and then the area can be obtained according to Heron's formula. The areas of $\Delta ABC$ and $\Delta ACD$ can be expressed as

$S_{\Delta ABC}=\frac{1}{2}\left| \vec{BA}\times\vec{BC} \right|$ (14)

and

$S_{\Delta ACD}=\frac{1}{2}\left| \vec{DA}\times\vec{DC} \right|$ (15)

1. **Additional details**
   1. ***Additional details of constructed random rough surfaces***

**a**

**b**

Fig. S1 The distribution of the area and roughness factor *R* of the structured rough surfaces with the same *R_q_.* **a** Area distribution of 10 structured rough surfaces through *R_q_* (2.0 $\mu m$, 3.0$\mu m$ and 5.0$\mu m$); **b** Roughness factor *R* of 10 structured rough surfaces through *R_q_* (2.0 $\mu m$, 3.0$\mu m$ and 5.0$\mu m$)

- 1. ***Additional details of relative increment of spectral-emissivity models caused by roughness***

**a**

**c**

**b**

**d**

Fig. S2 Relative increment of spectral-emissivity models caused by roughness. **a** Linear type spectral emissivity; **b** Quadratic type spectral emissivity; **c** Sinusoidal type spectral emissivity; **d** Exponential type spectral emissivity

- 1. ***Additional details of GH3044, K465, DD6, and TC4 alloys***

Table S1 Root-mean-square (RMS) deviation *R_q_* of GH3044, K465, DD6, and TC4 samples

| GH3044 | | | | K465 | | |
| --- | --- | --- | --- | --- | --- | --- |
|  | Sample index | Finishes | *R_q_* at 300 K（*μm*） | Sample index | Finishes | *R_q_* at 300 K（*μm*） |
| Reference | G0 | 1200-grit | 0.76 | K0 | 240-grit | 0.59 |
| Estimated | G1 | 80-grit | 2.10 | K1 | 36-grit | 2.39 |
| Estimated | G2 | 36-grit | 3.12 | K2 | unpolished | 3.70 |
| TC4 | | | | DD6 | | |
|  | Sample index | Finishes | *R_q_* at 300 K（*μm*） | Sample index | Finishes | *R_q_* at 300 K（*μm*） |
| Reference | T0 | 220-grit | 0.48 | D0 | 220-grit | 0.45 |
| Estimated | T1 | 100-grit | 2.41 | D1 | 100-grit | 2.34 |
| Estimated | T2 | 36-grit | 3.38 | D2 | unpolished | 3.60 |

- 1. ***Additional details of method implementation***


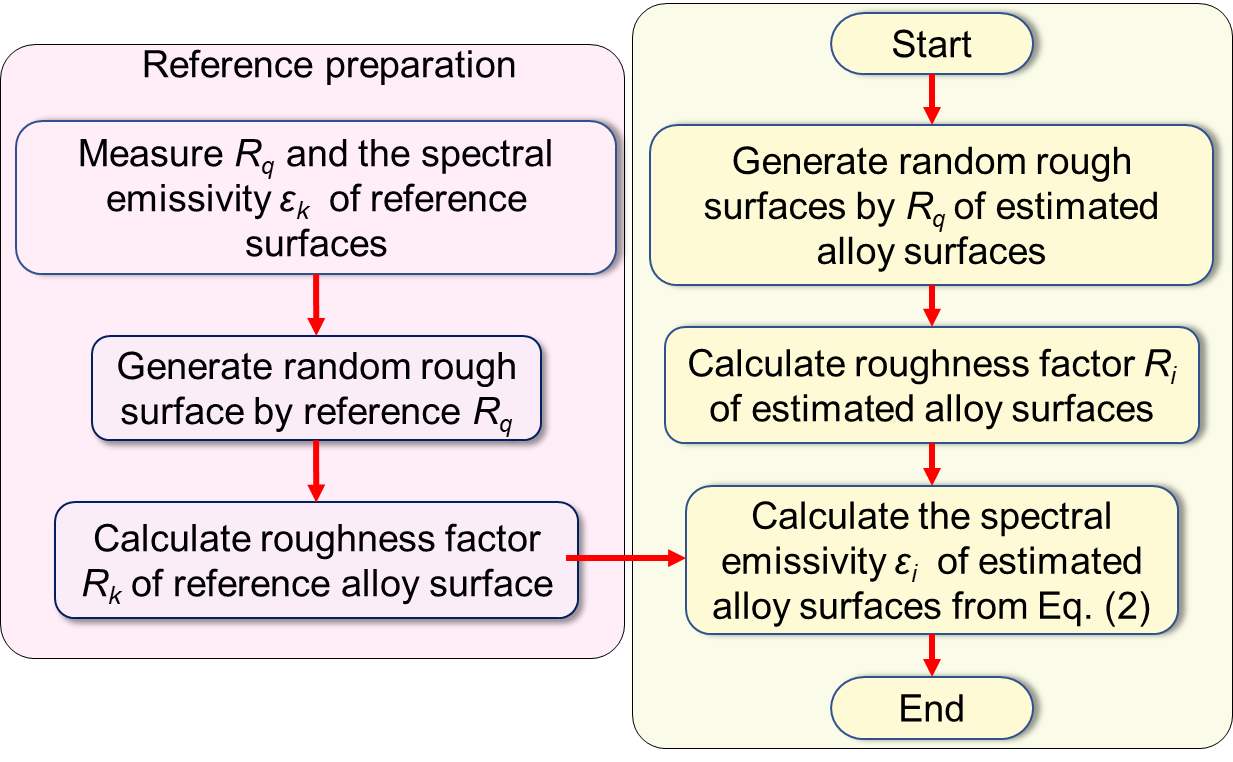


Fig. S3 Flow chart for estimating the spectral emissivity of the alloy surfaces

- 1. ***Additional details of result of calculating TC4 samples by proposed method***

d

b

c

a

**a**

**b**

**c**

**d**

Fig. S4 Spectral emissivity obtained by SEEM and measuring the TC4 alloy samples. **a** Spectral emissivity calculation and measurement results for sample T1; **b** Relative error in calculation and measurement of surface spectral emissivity for sample T1; **c** Spectral emissivity calculation and measurement results for sample T2; **d** Relative error in calculation and measurement of surface spectral emissivity for sample T2

- 1. ***Additional details of result of calculating DD6 samples by proposed method***

**a**

**b**

**c**

**d**

Fig. S5 Spectral emissivity obtained by SEEM and measuring the DD6 alloy samples. **a** Spectral emissivity calculation and measurement results for sample D1; **b** Relative error in calculation and measurement of surface spectral emissivity for sample D1; **c** Spectral emissivity calculation and measurement results for sample D2; **d** Relative error in calculation and measurement of surface spectral emissivity for sample D2

- 1. ***Additional details of result of calculating cobalt samples by proposed method***

**b**

**d**

**a**

**c**

Fig. S6 Spectral emissivity obtained by estimating and measuring cobalt samples at 773 K. **a** Spectral emissivity calculation and measurement results for sample 11; **b** Relative error in calculation and measurement of sample 11; **c** Spectral emissivity calculation and measurement results for sample 13; **d** Relative error in calculation and measurement of surface spectral emissivity for sample 13

- 1. ***Additional details of GH3044, K465, DD6, and TC4 samples***

Table S2 Element composition of GH3044, K465, DD6, and TC4 alloys at 300 K

| Content of element (at. %) | | | | | | | | | |
| --- | --- | --- | --- | --- | --- | --- | --- | --- | --- |
| GH3044 | Ni | Cr | W | Al | Si | Mn | Fe |  |  |
|  | Bal. | 23.5-26.5 | 13-16 | ≤0.5 | ≤0.8 | ≤0.5 | ≤4 |  |  |
| K465 | Ni | Cr | W | Al | Ti | Mo | Co | Nb | Zr |
|  | Bal. | 10 | 10.5 | 5.5 | 2.8 | 2.2 | 11 | 1.1 | ＜0.04 |
| TC4 | Ti | Al | Fe | C | N | H | O | V |  |
|  | Bal. | 6.06 | 0.15 | 0.02 | 0.02 | 0.006 | 0.17 | 4.03 |  |
| DD6 | Ni | Co | W | Al | Ta | Cr | Mo | Ti | B |
|  | Bal. | 8.5-9.5 | 7-9 | 5.2-6.2 | 6-8.5 | 5.2-6.2 | 1.5-2.5 | ≤0.1 | ≤0.02 |
|  | Fe | Mn |  |  |  |  |  |  |  |
|  | ≤0.2 | ≤0.15 |  |  |  |  |  |  |  |

**Supplementary Reference**

1 Tsz-King, C. *et al*. Experimental studies of bistatic scattering from two-dimensional conducting random rough surfaces. *IEEE Transactions on Geoscience and Remote Sensing* **34**, 674-680 (1996).

2 Zhou, W. *et al*. Modeling of rough surfaces with given roughness parameters. *Journal of Central South University* **24**, 127-136 (2017).
